# Effects of physical activity interventions on mental health in children and adolescents: a systematic review and meta-analysis of intervention type and baseline risk as moderators

**DOI:** 10.3389/fpsyg.2026.1825603

**Published:** 2026-05-29

**Authors:** Qian Liu, Zongyin Xu, Pengfei Tai, Zihao Lian, Weibo Cheng, Junping Zhang

**Affiliations:** 1 School of Sports Science, Qufu Normal University, Jining, China; 2 Shandong City Service Institute, Yantai, China; 3 Shandong Vocational College of Labor Technology, Jinan, China

**Keywords:** anxiety, children and adolescents, intervention type, mental health, meta-analysis, moderation analysis, physical activity

## Abstract

**Background:**

Mental health problems among children and adolescents are an increasing public health concern, and physical activity has attracted attention as a scalable non-pharmacological intervention. However, the effects of physical activity may vary according to intervention content and participant characteristics, and clearer evidence is needed to guide the selection and implementation of interventions.

**Methods:**

The protocol was registered in PROSPERO (CRD420251182579) and the review was conducted in accordance with PRISMA guidelines for systematic reviews and meta-analyses. The search was conducted in PubMed, Embase, PsycINFO, the Cochrane Library, Web of Science, and SPORTDiscus. Eligible studies included randomized controlled trials (RCTs) and quasi-experimental studies. Two reviewers independently performed study selection, data extraction, and risk-of-bias assessment. Standardized mean differences (SMDs) were pooled using a random-effects model, with prespecified subgroup, sensitivity, and publication-bias analyses.

**Results:**

A total of 24 studies were included in the qualitative synthesis, of which 23 contributed 38 effect-size estimates to the meta-analysis. Physical activity interventions were associated with a small but statistically significant improvement in mental health outcomes (SMD = 0.19, 95% CI: 0.09–0.30; *p* = 0.0002), with moderate heterogeneity (*I*^2^ = 48%; Q = 71.14; *p* = 0.0006). Intervention type significantly moderated the effect (Chi^2^ = 8.42; *p* = 0.04), with a relatively larger pooled estimate observed for mind–body interventions (SMD = 0.32, 95% CI: 0.15–0.49), followed by multicomponent/behavioral interventions (SMD = 0.18, 95% CI: 0.01–0.35), whereas the effects of sports/games and aerobic/fitness training were positive but not statistically significant. Baseline psychological risk status was also a significant moderator (Chi^2^ = 4.87; *p* = 0.03), with larger effects in high-risk/trait groups (SMD = 0.31, 95% CI: 0.14–0.48) than in the general population (SMD = 0.16, 95% CI: 0.07–0.25). Sensitivity analyses supported the robustness of the findings.

**Conclusion:**

Physical activity interventions were associated with small but significant improvements in mental health among children and adolescents. The findings suggest potentially larger benefits for mind–body interventions and for youth with elevated psychological risk, although these subgroup findings should be interpreted cautiously. These results support the targeted use of structured physical activity programs in school, public health, and early prevention settings.

**Systematic review registration:**

https://www.crd.york.ac.uk/PROSPERO/view/CRD420251182579, identifier: CRD420251182579.

## 1 Introduction

Mental health problems among children and adolescents represent an urgent and growing global public health burden. Anxiety and depressive disorders impose a substantial disease burden during this developmental period and may have lasting consequences for emotional development, social functioning, academic performance, and overall wellbeing (Kieling et al., 2024; Bie et al., 2024). Establishing effective, scalable, and acceptable early prevention and intervention strategies is therefore essential. In this context, physical activity has attracted considerable attention as a non-pharmacological intervention with multiple physiological and psychological benefits (Li et al., 2024). Observational evidence also supports its association with better mental health and psychological wellbeing among young people (Drapeau et al., 2025; Li et al., 2024).

Physical activity interventions for children and adolescents can take diverse forms, including structured exercise training, sports and games, school-based activity programs, mind–body practices, and multicomponent behavioral programs. These interventions may benefit mental health through physiological and psychosocial pathways, such as improved fitness, sleep, neuroendocrine regulation, stress responses, self-efficacy, perceived competence, social connectedness, emotional regulation, and positive mastery experiences. However, physical activity is a multidimensional intervention, and its effects may vary according to activity type, intervention content, participant characteristics, and the mental health constructs assessed (Teychenne et al., 2026). Therefore, pooling interventions with different active components, such as yoga and high intensity interval training, may obscure meaningful differences in mechanisms and practical effects.

Although previous systematic reviews have synthesized evidence on physical activity and mental health in youth, several limitations remain. Many reviews have treated heterogeneous physical activity interventions as a single construct or classified them using broad exercise physiology labels, such as aerobic vs. anaerobic activity, rather than differentiating interventions according to theoretical underpinnings and active components (Lu et al., 2025; White et al., 2024). In addition, key moderators, including developmental stage and baseline psychological risk status, have not been examined consistently or in sufficient depth (Pearce et al., 2022; Teychenne et al., 2026). Clarifying these issues is important for determining which types of physical activity interventions are most beneficial and for whom.

To address these gaps, we conducted a systematic review and meta-analysis of physical activity interventions for mental health outcomes in children and adolescents. We classified interventions into four prespecified categories: mind–body interventions, multicomponent or behavioral interventions, sports and games, and aerobic or fitness training. We further examined whether intervention effects differed by age group, baseline psychological risk status, and measurement characteristics. By incorporating recent evidence, including trials targeting adolescents with attention-deficit/hyperactivity disorder, this study aimed to provide more precise evidence to guide the selection and targeted implementation of physical activity interventions for youth mental health promotion and early prevention (Liu et al., 2025; da Silva et al., 2025).

## 2 Methods

We conducted a systematic review and meta-analysis to comprehensively evaluate the effects of physical activity interventions on mental health outcomes in children and adolescents, with anxiety symptoms as the primary focus. The protocol was registered in the International Prospective Register of Systematic Reviews (PROSPERO) (CRD420251182579). This report is prepared in accordance with the PRISMA 2020 statement.

### 2.1 Search strategy

We systematically searched six internationally recognized electronic bibliographic databases spanning medicine, psychology, sport science, and multidisciplinary research to maximize coverage: PubMed, Embase, PsycINFO, the Cochrane Library, Web of Science, and SPORTDiscus. The search covered each database from inception to February 28, 2026. The database searches were conducted without language restrictions; however, because of limited translation resources, only studies published in English or Chinese were eligible for inclusion. Two reviewers with systematic review experience independently developed and implemented the search strategy and cross-checked the search process. Disagreements were resolved through discussion and consensus.

The search strategy was developed using the PICOS framework (participants, interventions, comparators, outcomes, and study design). Participant-, intervention-, and outcome-related terms formed the core concepts and were searched using both controlled vocabulary (e.g., MeSH, Emtree, and database-specific thesauri) and free-text terms, combined with Boolean operators (AND, OR, and NOT).

To reduce publication bias and minimize database omissions, we conducted supplementary searches by screening the reference lists of included studies, reviewing the reference lists of relevant systematic reviews/meta-analyses published within the past 5 years, and searching gray literature sources, including ClinicalTrials.gov, WHO ICTRP, and ProQuest Dissertations & Theses Global. Relevant conference abstracts/proceedings were also considered if identified through these supplementary sources. Records identified through supplementary searches were screened using the same eligibility criteria as database records, but no additional eligible reports were identified. The complete search strategies for all databases and supplementary sources are provided in Supplementary Table 1.

### 2.2 Inclusion and exclusion criteria

In accordance with the prespecified protocol and the PICOS framework, we defined explicit inclusion and exclusion criteria to ensure that study selection was transparent, reproducible, and aligned with the review objectives. These criteria were designed to capture intervention evidence on physical activity and mental health in children and adolescents while balancing methodological rigor with the inclusion of relevant real-world evidence from quasi-experimental studies.

#### 2.2.1 Study design

We included randomized controlled trials (RCTs), cluster randomized controlled trials, and quasi-experimental studies published in peer-reviewed journals. Quasi-experimental studies were included to capture evidence from school- or community-based physical activity programs where full randomization may be infeasible. We excluded reviews, protocols, letters, case reports, observational studies, and other non-interventional designs.

#### 2.2.2 Participants

We included studies involving children and adolescents aged 3 to 19 years at intervention initiation, including both general population samples and risk or trait samples. Risk or trait samples included participants with psychosocial risk factors, such as low household income or ethnic minority status, or mild to moderate mental health problems, such as attention-deficit/hyperactivity disorder (ADHD) or subclinical anxiety or depressive symptoms. Studies primarily involving participants with severe physical illnesses requiring specialized medical care or severe psychiatric disorders requiring inpatient care were excluded.

#### 2.2.3 Interventions

We included structured physical activity programs designed to improve psychological outcomes through physical exercise. Interventions were classified *a priori* into four categories for subgroup analyses: mind–body interventions, sports/games interventions, multicomponent/behavioral interventions, and aerobic/fitness training.

Comparators included no intervention or wait-list control, usual care/standard curriculum, attention controls (such as health education), and psychological or behavioral interventions without a physical activity component.

#### 2.2.4 Outcomes

The primary outcome was mental health assessed using standardized and validated psychological scales. To enhance consistency across studies, we prespecified a hierarchy for outcome extraction, prioritizing anxiety-specific scales, followed by internalizing-problems subscales and broader measures of overall mental health, emotional distress, or positive affect. Post-intervention assessments conducted immediately after intervention completion were prioritized, and the latest available follow-up was additionally extracted when reported. Outcomes based solely on physiological indicators, academic performance, or non-validated measures were excluded.

#### 2.2.5 Exclusion criteria

In addition to the exclusions specified above, studies were excluded if the physical activity component could not be isolated from another active treatment, if the full text was unavailable, or if sufficient data to calculate effect sizes could not be obtained from the report or after contacting the authors.

### 2.3 Study selection and data extraction

Records were imported into reference-management software and deduplicated. Two reviewers independently screened titles/abstracts and full texts; disagreements were resolved through discussion and, when necessary, consultation with a third reviewer. The selection process and reasons for exclusion were documented using a PRISMA 2020 flow diagram.

Using a prespecified data-extraction form, two reviewers independently extracted study characteristics (first author, publication year, country, and study design), participant characteristics (sample size, age range or mean age, sex/gender composition, and baseline risk status), intervention and comparator characteristics (intervention type, frequency, intensity, duration, and comparator condition), and outcome information (instrument name and assessment time points). We prioritized data collected immediately post-intervention. Outcome data required for effect-size computation (means, standard deviations, and sample sizes) were extracted for eligible outcomes or comparisons.

### 2.4 Risk of bias assessment

Risk of bias was assessed using design-specific tools. Two reviewers independently assessed randomized controlled trials, including cluster randomized trials, using the Cochrane Risk of Bias tool version 2 (RoB 2). Quasi-experimental studies were assessed using ROBINS-I. Assessments were based on full texts and available supplementary materials. Disagreements were resolved through discussion and consensus, with a third reviewer consulted when necessary. Risk-of-bias judgments were summarized and visualized at the domain and overall levels and were used to inform the interpretation of the findings.

### 2.5 Statistical analysis

To ensure transparency and reproducibility of the quantitative synthesis, all statistical analyses were conducted according to a prespecified analysis plan. Effect-size estimates were calculated and pooled using standardized procedures. For each included study, we extracted post-intervention means, standard deviations, and sample sizes for the intervention and control groups for the primary mental health outcome. The primary effect measure was the standardized mean difference (SMD). We applied the Hedges' g small-sample correction to reduce small-sample bias. SMDs were oriented so that positive values favored the intervention and reflected better mental health, indicated by lower levels of anxiety or psychological distress. The primary synthesis combined anxiety-specific symptoms, internalizing problems, and emotional-state outcomes because these measures all reflect closely related dimensions of negative affect or psychological distress in children and adolescents. However, we recognized that these outcomes are not conceptually identical. Therefore, we conducted subgroup analyses by measurement type. When a study reported more than one eligible outcome measure at the same post-intervention time point, each was extracted as an effect-size estimate. Because multiple effect-size estimates from the same study may be statistically correlated, we did not treat them as fully independent. To examine whether within-study multiplicity influenced the pooled findings, we conducted a sensitivity analysis retaining one effect-size estimate per study. When multiple estimates were available within a study, the estimate with the greatest statistical precision was retained.

Effect-size estimates were pooled using a random-effects model (DerSimonian–Laird), which assumes that true effects vary across studies and provides an average overall estimate. We reported pooled SMDs with 95% confidence intervals (CIs) and *p* values. The *p* values for pooled subgroup effects are reported in the main text, while Tables 1–4 summarize pooled SMDs, 95% CIs, heterogeneity statistics, and subgroup-difference tests. Statistical significance of pooled effects was determined by jointly considering the 95% CI and *p* value; pooled effects were considered statistically significant when the 95% CI did not include 0 and the corresponding *p* value was < 0.05. Fixed-effect models were additionally used in sensitivity analyses to examine the robustness of results under alternative model assumptions. To further examine the robustness of the findings using a more conceptually homogeneous outcome category, we conducted an additional sensitivity analysis restricted to anxiety-specific scales. Between-study heterogeneity was assessed using the *I*^2^ statistic and Cochran's *Q* test. According to the Cochrane Handbook, heterogeneity was considered statistically significant when *I*^2^ > 50% or *p* < 0.10.

**Table 1:** Study-specific effect sizes and pooled results of subgroup analyses by intervention type.

Intervention type	Study (year)	SMD (95% CI)
Mind–body interventions		0.32 [0.15, 0.49]
	Bazzano et al. (2022)a	0.24 [−0.43, 0.91]
	Bazzano et al. (2022)b	0.50 [0.00, 1.00]
	Khalsa et al. (2012)	0.18 [−0.16, 0.52]
	(Lobo and Winsler 2006)	0.08 [−1.68, 1.84]
	Noggle et al. (2012)a	0.97 [0.21, 1.73]
	Noggle et al. (2012)b	0.34 [−0.34, 1.02]
	Noggle et al. (2012)c	0.28 [−0.42, 0.98]
	Pradhan and Pramanik (2024)a	0.98 [0.03, 1.93]
	Pradhan and Pramanik (2024)b	0.98 [0.51, 1.45]
	Quach et al. (2016)a	0.05 [−0.34, 0.44]
	Quach et al. (2016)b	−0.04 [−0.85, 0.77]
	Telles et al. (2013)	0.98 [0.35, 1.61]
Heterogeneity	*I^2^* = 45%, *P* = 0.03	
Multicomponent/behavioral interventions		0.18 [0.01, 0.35]
	Ardic and Erdogan (2017)a	0.17 [−0.38, 0.72]
	Ardic and Erdogan (2017)b	0.26 [−0.37, 0.89]
	Bohnert and Ward (2013)a	0.02 [−0.45, 0.49]
	Bohnert and Ward (2013)b	0.12 [−0.32, 0.56]
	Melnyk et al. (2009)a	−0.25 [−0.87, 0.37]
	Melnyk et al. (2009)b	0.13 [−0.61, 0.87]
	Smith et al. (2018)	0.16 [0.03, 0.29]
Heterogeneity	*I^2^* = 30%, *P* = 0.18	
Sports/Games interventions		0.09 [−0.05, 0.23]
	Barton et al. (2015)	−0.44 [−0.77, −0.11]
	Cocca et al. (2020)a	−0.02 [−0.33, 0.29]
	Cocca et al. (2020)b	0.03 [−0.41, 0.47]
	Cocca et al. (2020)c	0.09 [−0.35, 0.53]
	Koszałka-Silska et al. (2021)a	0.15 [−0.32, 0.62]
	Koszałka-Silska et al. (2021)b	−0.40 [−0.87, 0.07]
	Ozyurek et al. (2015)	0.43 [−0.29, 1.15]
	Xu et al. (2021)	0.49 [−0.09, 1.07]
Heterogeneity	*I^2^* = 25%, *P* = 0.21	
Aerobic/fitness training		0.05 [−0.10, 0.20]
	Burgess et al. (2006) Crews et al. (2004)a	0.23 [0.06, 0.40] 0.44 [−0.10, 0.98]
	Crews et al. (2004)b	0.43 [−0.11, 0.97]
	Crews et al. (2004)c	0.90 [0.42, 1.38]
	Cowley et al. (2021)	0.45 [−0.04, 0.94]
	da Silva et al. (2025)a	0.22 [−0.01, 0.45]
	da Silva et al. (2025)b	0.02 [−0.20, 0.24]
	Eather et al. (2016)	−0.04 [-0.58, 0.50]
	Liu et al. (2025)a	−0.54 [−1.66, 0.58]
	Liu et al. (2025)b	−0.59 [−1.84, 0.66]
	Wassenaar et al. (2021)	−0.07 [−0.61, 0.47]
Heterogeneity	*I^2^* = 15%, *P* = 0.30	
Test for subgroup differences	Chi^2^ = 8.42, df = 3, *P* = 0.04	

^a, b, c^ indicate different effect-size estimates from the same study.

**Table 2:** Subgroup analysis results stratified by participant age/stage.

Age/stage subgroup	Study (year)	SMD (95% CI)
Children ( ≤ 12 years)		0.12 [0.02, 0.22]
	Barton et al. (2015)	−0.44 [−0.77, −0.11]
	Bohnert and Ward (2013)a	0.02 [−0.45, 0.49]
	Bohnert and Ward (2013)b	0.12 [−0.32, 0.56]
	Cocca et al. (2020)a	−0.02 [−0.33, 0.29]
	Cocca et al. (2020)b	0.03 [−0.41, 0.47]
	Cocca et al. (2020)c	0.09 [−0.35, 0.53]
	Crews et al. (2004)a	0.44 [−0.10, 0.98]
	Crews et al. (2004)b	0.43 [−0.11, 0.97]
	Crews et al. (2004)c	0.90 [0.42, 1.38]
	da Silva et al. (2025)a	0.22 [−0.01, 0.45]
	da Silva et al. (2025)b	0.02 [−0.20, 0.24]
	Lobo and Winsler (2006)	0.08 [−1.68, 1.84]
	Ozyurek et al. (2015)	0.43 [−0.29, 1.15]
	Telles et al. (2013)	0.98 [0.35, 1.61]
Heterogeneity	I^2^ = 35%, P = 0.08	
Adolescents (> 12 years)		0.25 [0.11, 0.39]
	Ardic and Erdogan (2017)a	0.17 [−0.38, 0.72]
	Ardic and Erdogan (2017)b	0.26 [−0.37, 0.89]
	Bazzano et al. (2022)a	0.24 [−0.43, 0.91]
	Bazzano et al. (2022)b	0.50 [0.00, 1.00]
	Burgess et al. (2006)	0.23 [0.06, 0.40]
	Cowley et al. (2021)	0.45 [−0.04, 0.94]
	Eather et al. (2016)	−0.04 [−0.58, 0.50]
	Khalsa et al. (2012)	0.18 [−0.16, 0.52]
	Koszałka-Silska et al. (2021)a	0.15 [−0.32, 0.62]
	Koszałka-Silska et al. (2021)b	−0.40 [−0.87, 0.07]
	Liu et al. (2025)a	−0.54 [−1.66, 0.58]
	Liu et al. (2025)b	−0.59 [−1.84, 0.66]
	Melnyk et al. (2009)a	−0.25 [−0.87, 0.37]
	Melnyk et al. (2009)b	0.13 [−0.61, 0.87]
	Noggle et al. (2012)a	0.97 [0.21, 1.73]
	Noggle et al. (2012)b	0.34 [−0.34, 1.02]
	Noggle et al. (2012)c	0.28 [−0.42, 0.98]
	Pradhan and Pramanik (2024)a	0.98 [0.03, 1.93]
	Pradhan and Pramanik (2024)b	0.98 [0.51, 1.45]
	Quach et al. (2016)a	0.05 [−0.34, 0.44]
	Quach et al. (2016)b	−0.04 [−0.85, 0.77]
	Smith et al. (2018)	0.16 [0.03, 0.29]
	Wassenaar et al. (2021)	−0.07 [−0.61, 0.47]
	Xu et al. (2021)	0.49 [−0.09, 1.07]
Heterogeneity	I^2^ = 40%, *P* = 0.01	
Test for subgroup differences	Chi^2^ = 1.95, df = 1, *P* = 0.16	

^a, b, c^ indicate different effect-size estimates from the same study.

**Table 3:** Subgroup analysis results by baseline risk status.

Risk-status subgroup	Study (year)	SMD (95% CI)
General population		0.16 [0.07, 0.25]
	Ardic and Erdogan (2017)a	0.17 [−0.38, 0.72]
	Ardic and Erdogan (2017)b	0.26 [−0.37, 0.89]
	Barton et al. (2015)	−0.44 [−0.77,−0.11]
	Bazzano et al. (2022)a	0.24 [−0.43, 0.91]
	Bazzano et al. (2022)b	0.50 [0.00, 1.00]
	Burgess et al. (2006)	0.23 [0.06, 0.40]
	Cocca et al. (2020)a	−0.02 [−0.33, 0.29]
	Cocca et al. (2020)b	0.03 [−0.41, 0.47]
	Cocca et al. (2020)c	0.09 [−0.35, 0.53]
	Cowley et al. (2021)	0.45 [−0.04, 0.94]
	Eather et al. (2016)	−0.04 [−0.58, 0.50]
	Khalsa et al. (2012)	0.18 [−0.16, 0.52]
	Koszałka-Silska et al. (2021)a	0.15 [−0.32, 0.62]
	Koszałka-Silska et al. (2021)b	−0.40 [−0.87, 0.07]
	Noggle et al. (2012)a	0.97 [0.21, 1.73]
	Noggle et al. (2012)b	0.34 [−0.34, 1.02]
	Noggle et al. (2012)c	0.28 [−0.42, 0.98]
	Ozyurek et al. (2015)	0.43 [−0.29, 1.15]
	Pradhan and Pramanik (2024)a	0.98 [0.03, 1.93]
	Pradhan and Pramanik (2024)b	0.98 [0.51, 1.45]
	Quach et al. (2016)a	0.05 [−0.34, 0.44]
	Quach et al. (2016)b	−0.04 [−0.85, 0.77]
	Smith et al. (2018)	0.16 [0.03, 0.29]
	Telles et al. (2013)	0.98 [0.35, 1.61]
	Wassenaar et al. (2021)	−0.07 [−0.61, 0.47]
	Xu et al. (2021)	0.49 [−0.09, 1.07]
Heterogeneity	*I^2^* = 35%, *P* = 0.02	
Risk/trait population		0.31 [0.14, 0.48]
	Bohnert and Ward (2013)a	0.02 [−0.45, 0.49]
	Bohnert and Ward (2013)b	0.12 [−0.32, 0.56]
	Crews et al. (2004)a	0.44 [−0.10, 0.98]
	Crews et al. (2004)b	0.43 [−0.11, 0.97]
	Crews et al. (2004)c	0.90 [0.42, 1.38]
	da Silva et al. (2025)a	0.22 [−0.01, 0.45]
	da Silva et al. (2025)b	0.02 [−0.20, 0.24]
	Liu et al. (2025)a	−0.54 [−1.66, 0.58]
	Liu et al. (2025)b	−0.59 [−1.84, 0.66]
	Lobo and Winsler (2006)	0.08 [−1.68, 1.84]
	Melnyk et al. (2009)a	−0.25 [−0.87, 0.37]
Melnyk et al. (2009)b	0.13 [−0.61, 0.87]
Heterogeneity	*I^2^* = 38%, *P* = 0.09	
Test for subgroup differences	Chi^2^ = 4.87, df = 1, *P* = 0.03	

^a, b, c^ indicate different effect-size estimates from the same study.

**Table 4:** Subgroup analysis results by measurement instrument type.

Measurement instrument subgroup	Study (year)	SMD (95% CI)
Anxiety-specific scales		0.22 [0.08, 0.36]
	Ardic and Erdogan (2017)a	0.17 [−0.38, 0.72]
	Ardic and Erdogan (2017)b	0.26 [−0.37, 0.89]
	Bazzano et al. (2022)a	0.24 [−0.43, 0.91]
	Bazzano et al. (2022)b	0.50 [0.00, 1.00]
	Cocca et al. (2020)a	−0.02 [−0.33, 0.29]
	Cocca et al. (2020)b	0.03 [−0.41, 0.47]
	Cocca et al. (2020)c	0.09 [−0.35, 0.53]
	Crews et al. (2004)a	0.44 [−0.10, 0.98]
	Crews et al. (2004)b	0.43 [−0.11, 0.97]
	Crews et al. (2004)c	0.90 [0.42, 1.38]
	da Silva et al. (2025)a	0.22 [−0.01, 0.45]
	da Silva et al. (2025)b	0.02 [−0.20, 0.24]
	Khalsa et al. (2012)	0.18 [−0.16, 0.52]
	Liu et al. (2025)a	−0.54 [−1.66, 0.58]
	Liu et al. (2025)b	−0.59 [−1.84, 0.66]
	Melnyk et al. (2009)a	−0.25 [−0.87, 0.37]
	Melnyk et al. (2009)b	0.13 [−0.61, 0.87]
	Pradhan and Pramanik (2024)a	0.98 [0.03, 1.93]
	Pradhan and Pramanik (2024)b	0.98 [0.51, 1.45]
	Quach et al. (2016)a	0.05 [−0.34, 0.44]
	Quach et al. (2016)b	−0.04 [−0.85, 0.77]
Heterogeneity	*I^2^* = 43.9%, *P* = 0.017	
Internalizing-problems subscales		0.01 [−0.17, 0.20]
	Barton et al. (2015)	−0.44 [−0.77, −0.11]
	Bohnert and Ward (2013)a	0.02 [−0.45, 0.49]
	Bohnert and Ward (2013)b	0.12 [−0.32, 0.56]
	Eather et al. (2016)	−0.04 [−0.58, 0.50]
	Koszałka-Silska et al. (2021)a	0.15 [−0.32, 0.62]
	Koszałka-Silska et al. (2021)b	−0.40 [−0.87, 0.07]
	Lobo and Winsler (2006)	0.08 [−1.68, 1.84]
	Ozyurek et al. (2015)	0.43 [−0.29, 1.15]
	Smith et al. (2018)	0.16 [0.03, 0.29]
	Wassenaar et al. (2021)	−0.07 [−0.61, 0.47]
	Xu et al. (2021)	0.49 [−0.09, 1.07]
Heterogeneity	*I^2^* = 46.4%, *P* = 0.045	
Emotional-state scales		0.46 [0.19, 0.72]
	Burgess et al. (2006)	0.23 [0.06, 0.40]
	Cowley et al. (2021)	0.45 [−0.04, 0.94]
	Noggle et al. (2012)a	0.97 [0.21, 1.73]
	Noggle et al. (2012)b	0.34 [−0.34, 1.02]
	Noggle et al. (2012)c	0.28 [−0.42, 0.98]
	Telles et al. (2013)	0.98 [0.35, 1.61]
Heterogeneity	I^2^ = 40.4%, *P* = 0.136	
Test for subgroup differences	Chi^2^ = 7.65, df = 2, *P* = 0.0218	

^a, b, c^ indicate different effect-size estimates from the same study.

### 2.6 Subgroup analysis

To explore potential sources of heterogeneity, we prespecified and conducted subgroup analyses. Differences between subgroups were tested under a random-effects model, with *p* < 0.05 indicating statistical significance. Subgroups were defined according to: (1) intervention characteristics, classified *a priori* into mind–body, multicomponent/behavioral, sports/games, and aerobic/fitness training interventions; (2) participant characteristics, including age group (children ≤ 12 years; adolescents > 12 years) and baseline psychological risk status (general-population vs. risk/trait samples); and (3) measurement characteristics, based on the construct assessed: anxiety-specific scales, internalizing-problems subscales, and emotional-state scales.

### 2.7 Publication bias and sensitivity analyses

For outcomes with at least 10 studies, we assessed potential publication bias using complementary approaches. We first visually inspected funnel plots by plotting effect sizes (SMDs) against their standard errors to evaluate small-study effects and asymmetry. We then applied Egger's regression test to quantify funnel-plot asymmetry, with a non-zero intercept indicating potential small-study effects. In addition, we used the trim-and-fill method under a random-effects model to estimate the number of potentially missing studies and to examine the influence of such missingness on the pooled estimate.

To assess the robustness of the pooled results, we conducted sensitivity analyses by performing leave-one-out analyses, in which the meta-analysis was repeated after omitting each study in turn, and by comparing pooled estimates derived from random-effects and fixed-effect models to evaluate the impact of model assumptions. All analyses were performed using RevMan 5.4 and R.

## 3 Results

### 3.1 Search results

**Figure 1:**
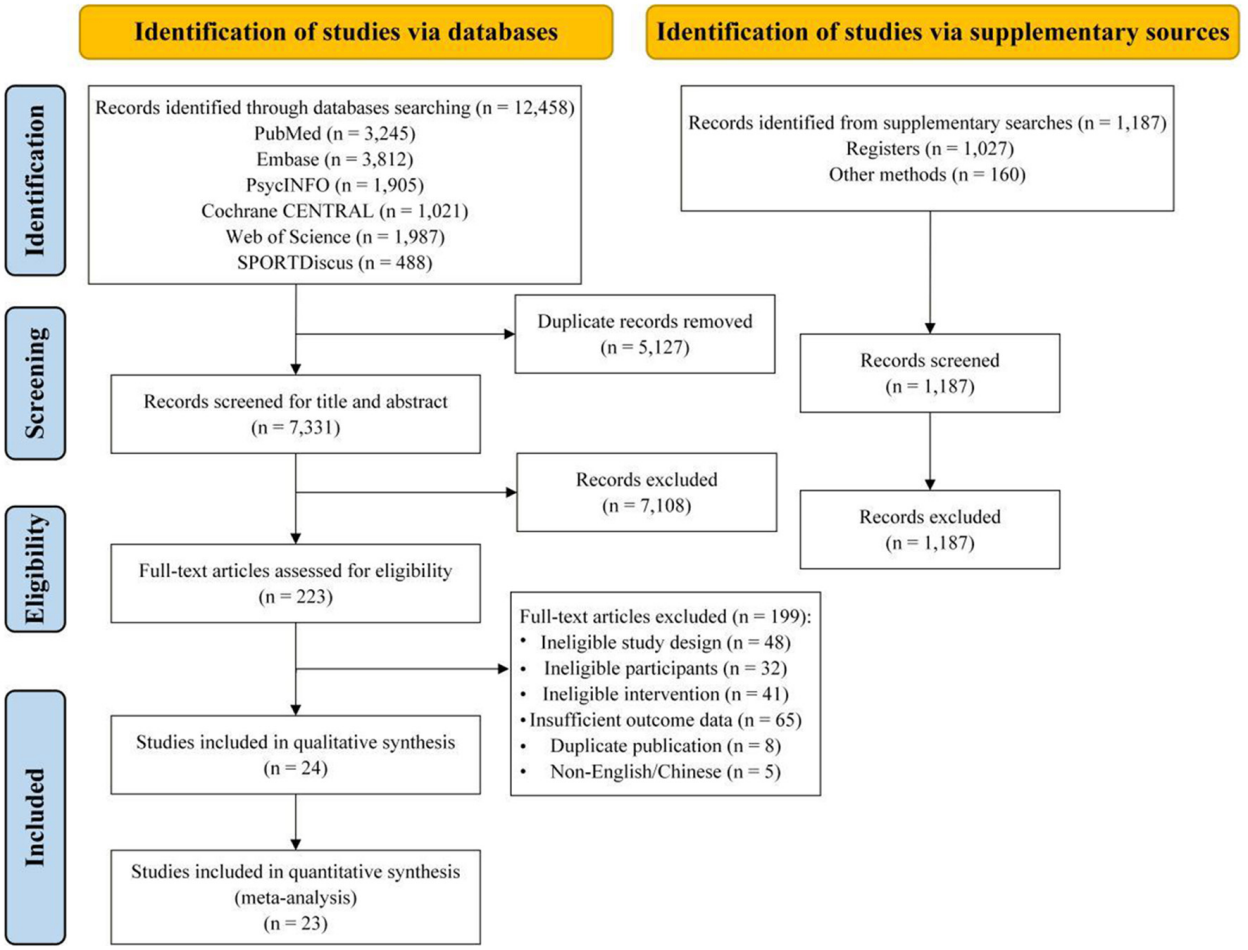
PRISMA flowchart.

Following the PRISMA statement guidelines, we conducted literature screening and selection. Through systematic searches of PubMed (*n* = 3,245), Embase (*n* = 3,812), PsycINFO (*n* = 1,905), Cochrane CENTRAL (*n* = 1,021), Web of Science (*n* = 1,987), and SPORTDiscus (*n* = 488), a total of 12,458 records were retrieved. After removing 5,127 duplicate records, 7,331 unique records remained for title and abstract screening. Two reviewers independently screened these records and excluded 7,108 as clearly irrelevant. The main reasons for exclusion included studies that did not involve children or adolescents, studies with ineligible designs such as cross-sectional surveys or reviews, studies with interventions that were not structured physical activity programs, and studies with topics not aligned with the review aims, including those focusing only on physiological outcomes or academic performance. In addition, 1,187 records were identified from supplementary sources, including trial registers (ClinicalTrials.gov and WHO ICTRP; *n* = 1,027) and other methods (citation searching of included studies and recent relevant systematic reviews/meta-analyses, and ProQuest Dissertations and Theses Global; *n* = 160). These records were screened using the same eligibility criteria as database records, but no additional eligible reports were identified. Full texts of the remaining 223 articles were retrieved and assessed for eligibility, resulting in the exclusion of 199 articles. Reasons for full-text exclusion were ineligible study design (*n* = 48), ineligible participants (*n* = 32), ineligible interventions (*n* = 41), insufficient outcome data for effect-size calculation (*n* = 65), duplicate or follow-up reports (*n* = 8), and publication in languages other than Chinese or English (*n* = 5). Ultimately, 24 studies met the inclusion criteria and were included in the qualitative synthesis. Of these, 23 studies provided sufficient mental health outcome data for quantitative synthesis, contributing 38 effect-size estimates. Figure 1 illustrates the detailed literature screening process.

### 3.2 Study characteristics

The included studies spanned more than two decades (2004–2025). More than half of the studies were published in the last decade (2016–2025; *n* = 13), and a substantial proportion were published in recent years (2020–2025; *n* = 9), indicating sustained research interest. Studies were conducted in 10 countries, most commonly the United States (*n* = 8, 33.3%) and the United Kingdom (*n* = 4, 16.7%). Other studies were conducted in countries including Australia, Turkey, India, China, and South Korea, indicating geographic diversity. Regarding study design, randomized controlled trials (RCTs) predominated (*n* = 18, 75%), including six cluster RCTs; the remainder comprised quasi-experimental studies (*n* = 5, 20.8%) and one study with another design. Sample sizes ranged from 19 to 16,017, and most studies (n = 18) enrolled fewer than 100 participants. Participant ages ranged from preschool (3–5 years) to late adolescence (15–19 years), with most studies focusing on middle-school–aged participants (11–16 years; *n* = 11). Regarding sex/gender composition, 19 studies (79.2%) included both females and males, three (12.5%) included females only, and two (8.3%) included males only. Most studies targeted the general student population, whereas several studies focused on at-risk/trait samples, including low-income or ethnic-minority samples and adolescents with ADHD.

### 3.3 Study quality and risk of bias

**Figure 2:**
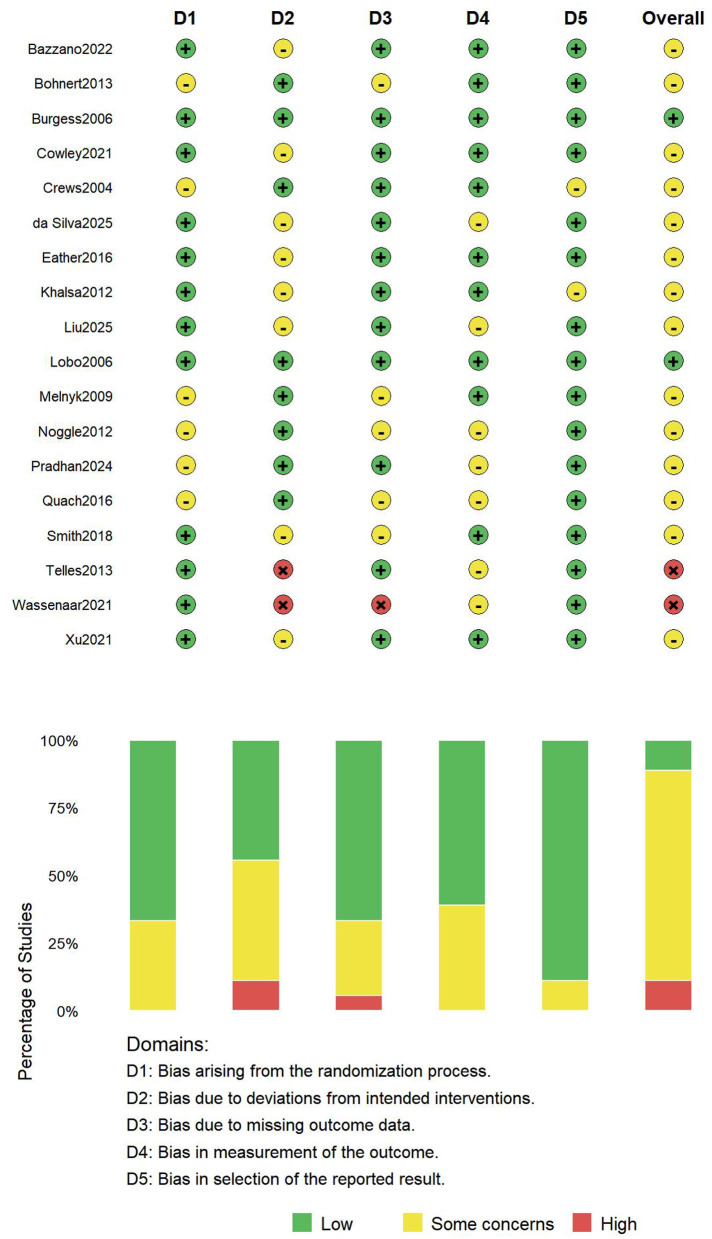
ROB2.

Risk of bias in the included randomized controlled trials was assessed using the Cochrane Risk of Bias tool, version 2 (RoB 2), and quasi-experimental studies were assessed using ROBINS-I. Figure 2 presents both a traffic-light plot and a summary plot showing the distribution of judgments across the RoB 2 domains and overall. Across the randomized trials, risk of bias was generally low for Domain 1 and Domain 5 indicating that most studies reported acceptable randomization procedures and did not raise major concerns about selective reporting. Performance was also relatively favorable for Domain 3, with most trials judged at low risk and only a small number showing concerns or high risk. In contrast, the most prominent concerns were observed in Domain 2, which relates to deviations from intended interventions, and to a lesser extent in Domain 4, which concerns outcome measurement. This pattern is consistent with the practical difficulty of blinding participants and intervention personnel in behavioral and exercise-based interventions and with the reliance on self-reported mental health measures in many trials, both of which can increase susceptibility to deviations from protocol and measurement-related bias. Overall, most randomized trials were rated as “some concerns,” with high risk of bias identified in only a small number of studies, such as Telles et al. (2013) and Wassenaar et al. (2021), primarily driven by concerns related to deviations from intended interventions and, in some cases, missing outcome data. For quasi-experimental studies, ROBINS-I assessments indicated an overall moderate risk of bias, with confounding and selection of participants emerging as the main sources of concern. Detailed domain-level ROBINS-I judgments for quasi-experimental studies are presented in Supplementary Figure 1.

### 3.4 Meta analysis overall effect

**Figure 3:**
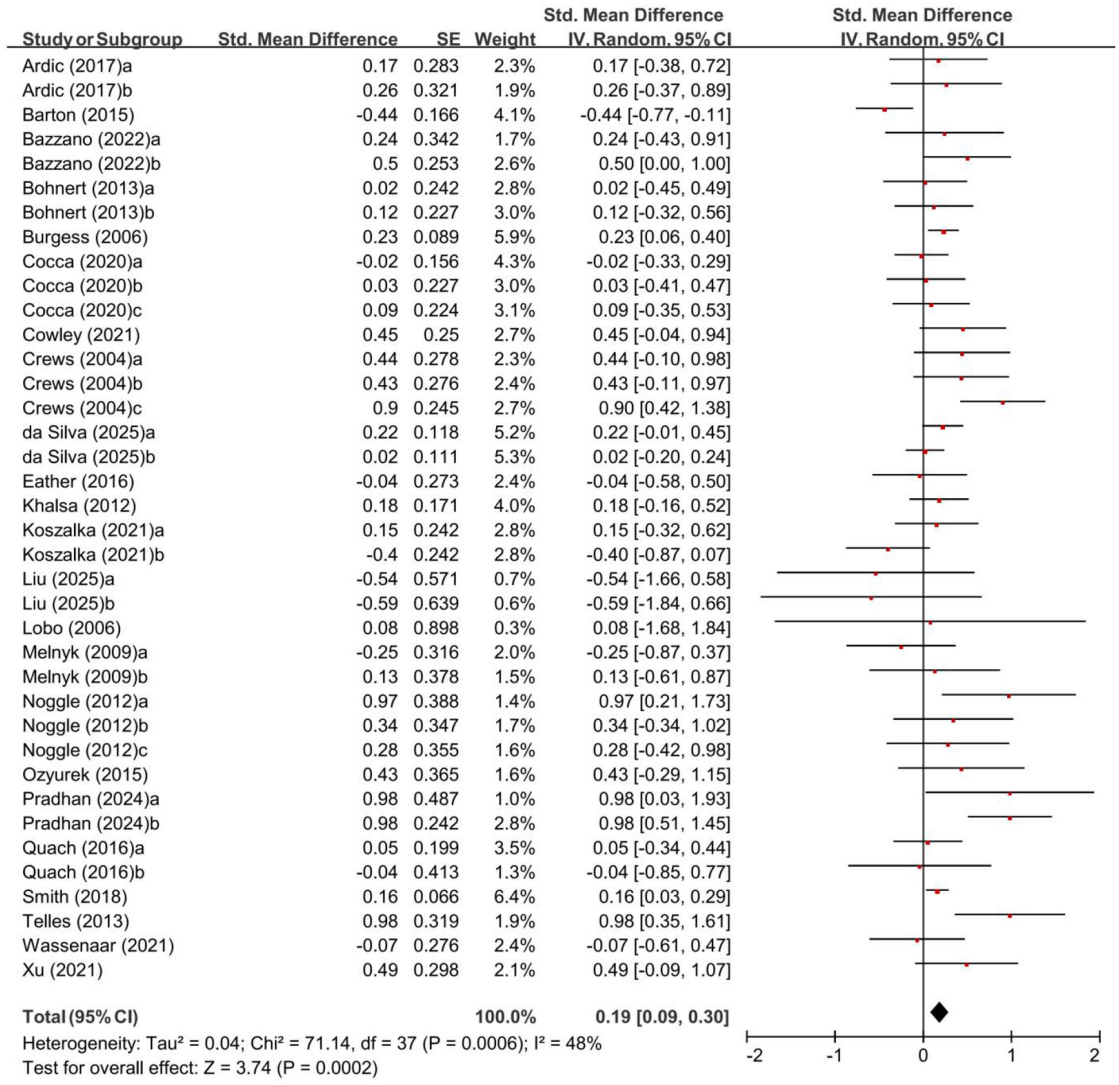
Forest plot.

Overall, 38 effect-size estimates were synthesized using a random-effects model, with standardized mean differences (SMDs) and 95% confidence intervals (CIs) used as the summary effect measures. The pooled effect showed that physical activity interventions were associated with a statistically significant improvement in mental health (SMD = 0.19, 95% CI: 0.09–0.30; *Z* = 3.74, *p* = 0.0002). Heterogeneity analyses indicated moderate between-study variability (*I*^2^ = 48%), and Cochran's *Q* test suggested statistically significant heterogeneity (*Q* = 71.14, df = 37, *p* = 0.0006), implying that true effects likely differ across studies. Accordingly, the use of a random-effects model was appropriate, and potential sources of heterogeneity were further examined in subgroup analyses. The forest plot (Figure 3) illustrates the distribution of individual effect-size estimates and their 95% CIs. For most comparisons, the 95% CI crossed the line of no effect (SMD = 0), indicating that many single studies were not statistically significant when considered in isolation. However, the pooled estimate lay entirely to the right of the line of no effect and its 95% CI did not include zero, demonstrating a statistically significant overall benefit after synthesizing evidence across studies. Effect-size estimates also varied notably across studies: Pradhan and Pramanik (2024) and Telles et al. (2013) reported comparatively large positive effects (SMD > 0.8), whereas Barton et al. (2015) showed a small effect in the negative direction. Overall, these results indicate that physical activity interventions confer a modest but reliable improvement in mental health among children and adolescents, while also highlighting meaningful variability in effects across studies. As an additional robustness check addressing within-study multiplicity, the one-estimate-per-study sensitivity analysis yielded a similar pooled effect (SMD = 0.20, 95% CI: 0.07–0.34; *p* = 0.0026), suggesting that the overall finding was not materially affected by retaining only one effect-size estimate per study (Supplementary Figure 2).

### 3.5 Subgroup analysis

#### 3.5.1 Effect differences based on intervention characteristics

To examine whether intervention type moderated between-study heterogeneity, we classified the included effect-size estimates into four prespecified, mutually exclusive subgroups based on intervention content, theoretical underpinnings, and primary activity format. Mind–body interventions included programs such as yoga, mindfulness-based meditation or movement, and creative dance, which typically integrate posture-based practice, breath regulation, meditative attention, and imagery to enhance interoceptive awareness and emotional self-regulation. Sports/games interventions included programs such as game-based physical education, adventure-based education, playground free-play activities, and novel sports, with an emphasis on engagement, skill challenge, social interaction, and teamwork rather than explicit psychological skill training. Multicomponent/behavioral interventions referred to structured programs that combined physical activity with additional components such as cognitive-behavioral skills, stress-management training, nutrition education, or leadership development, thereby influencing mental health through multiple cognitive, behavioral, and lifestyle pathways. Aerobic/fitness training comprised structured training primarily targeting cardiorespiratory fitness or muscular strength, including aerobic dance, high-intensity interval training, resistance training such as CrossFit™ Teens, and traditional aerobic circuit training. Details of the studies included in each subgroup and their effect sizes are presented in Table 1. Subgroup analyses were conducted using a random-effects model, and between-subgroup differences were evaluated using the χ^2^ test for subgroup differences.

Mind–body interventions showed a relatively larger pooled estimate (SMD = 0.32, 95% CI: 0.15–0.49; *p* < 0.001), suggesting a potentially greater effect relative to the other intervention categories. Multicomponent/behavioral interventions also showed a small but statistically significant positive effect (SMD = 0.18, 95% CI: 0.01–0.35; *p* = 0.038). In contrast, the pooled effects for sports/games interventions (SMD = 0.09, 95% CI: −0.05–0.23; *p* = 0.208) and aerobic/fitness training (SMD = 0.05, 95% CI: −0.10–0.20; *p* = 0.514) were positive but not statistically significant because their confidence intervals included 0. Within subgroups, heterogeneity was reduced relative to the overall analysis, with *I*^2^ values ranging from 15% to 45% compared with an overall *I*^2^ of 48%. Heterogeneity tests were not statistically significant for most subgroups, except for the mind–body subgroup, suggesting residual differences in intervention content, duration, or implementation fidelity across programs within this category. The test for subgroup differences was statistically significant (*p* = 0.04), providing evidence that intervention type is an important moderator of the effects of physical activity on mental health.

#### 3.5.2 Effect differences based on participant characteristics

To examine whether intervention effects varied by participant characteristics, we conducted prespecified subgroup analyses by age/stage and baseline psychological risk status. Subgroup analyses suggested that intervention effects differed by baseline risk status, with larger benefits observed in risk/trait groups. Although pooled effects were numerically larger in adolescents, the test for subgroup differences by age/stage was not statistically significant. These findings highlight the potential value of prioritizing youth with elevated psychological risk when designing and implementing future programs to maximize public health impact.

(1) Age/stage subgroup

Based on psychosocial developmental stages and typical schooling structure, participants were grouped into children ( ≤ 12 years) and adolescents (> 12 years). The pooled effect was larger in adolescents (SMD = 0.25, 95% CI: 0.11–0.39; *p* < 0.001) than in children (SMD = 0.12, 95% CI: 0.02–0.22; *p* = 0.019), suggesting a potentially greater average benefit in adolescents. Confidence intervals for both subgroups excluded 0, indicating statistically significant effects in each age group. However, the test for subgroup differences was not statistically significant (p = 0.16), indicating that the observed difference may reflect sampling variation rather than a definitive age-related moderating effect (Table 2).

#### 3.5.3 Baseline risk-status subgroup

Participants were grouped according to baseline psychosocial risk status. General-population samples were recruited from typical school settings and were not selected based on psychological symptoms or elevated risk. Risk/trait samples were defined by clear risk characteristics, including low socioeconomic status, a diagnosis of ADHD, or elevated baseline psychological distress. The pooled effect was larger in risk/trait samples (SMD = 0.31, 95% CI: 0.14–0.48; *p* < 0.001) than in general-population samples (SMD = 0.16, 95% CI: 0.07–0.25; *p* < 0.001), and the test for subgroup differences was statistically significant (*p* = 0.03) (Table 3). This suggests that physical activity interventions may yield greater mental health benefits among youth with psychosocial risk factors or mild symptoms at baseline. These findings underscore the potential value of targeted prevention and early intervention.

#### 3.5.4 Effect differences based on measurement characteristics

To examine whether measurement instruments influenced effect estimates, we stratified effect sizes into three subgroups based on the construct assessed by the scale: anxiety-specific scales, internalizing-problems subscales, and emotional-state scales. Anxiety-specific scales were standardized instruments designed to assess anxiety symptoms, including the Beck Anxiety Inventory (BAI) and the Screen for Child Anxiety Related Emotional Disorders (SCARED). Internalizing-problems subscales were drawn from broader mental health or behavioral measures and typically do not distinguish anxiety from other internalizing symptoms, such as depression and withdrawal; examples included SDQ and SCBE internalizing scores. Emotional-state scales captured transient affect, typically using a tension/anxiety subscale, such as the POMS-SF Tension–Anxiety subscale.

As shown in Table 4, the emotional-state scale subgroup showed the largest pooled estimate (SMD = 0.46, 95% CI: 0.19–0.72; *p* < 0.001), followed by the anxiety-specific scale subgroup (SMD = 0.22, 95% CI: 0.08–0.36; *p* = 0.0017). In contrast, the internalizing-problems subgroup showed a smaller and statistically non-significant pooled effect (SMD = 0.01, 95% CI: −0.17–0.20; *p* = 0.885). The sensitivity analysis restricted to anxiety-specific scales yielded a positive and statistically significant effect (SMD = 0.22, 95% CI: 0.08–0.36; *p* = 0.0017; Supplementary Figure 3). Heterogeneity was moderate across the three measurement-type subgroups. The test for subgroup differences was statistically significant (Chi^2^ = 7.65, df = 2, *p* = 0.0218), suggesting that pooled estimates varied across measurement types. However, because the number of effect-size estimates was limited in some subgroups and between-study variability remained, these measurement-type subgroup findings should be interpreted cautiously.

### 3.6 Sensitivity analysis

#### 3.6.1 Leave-one-out analysis

**Figure 4:**
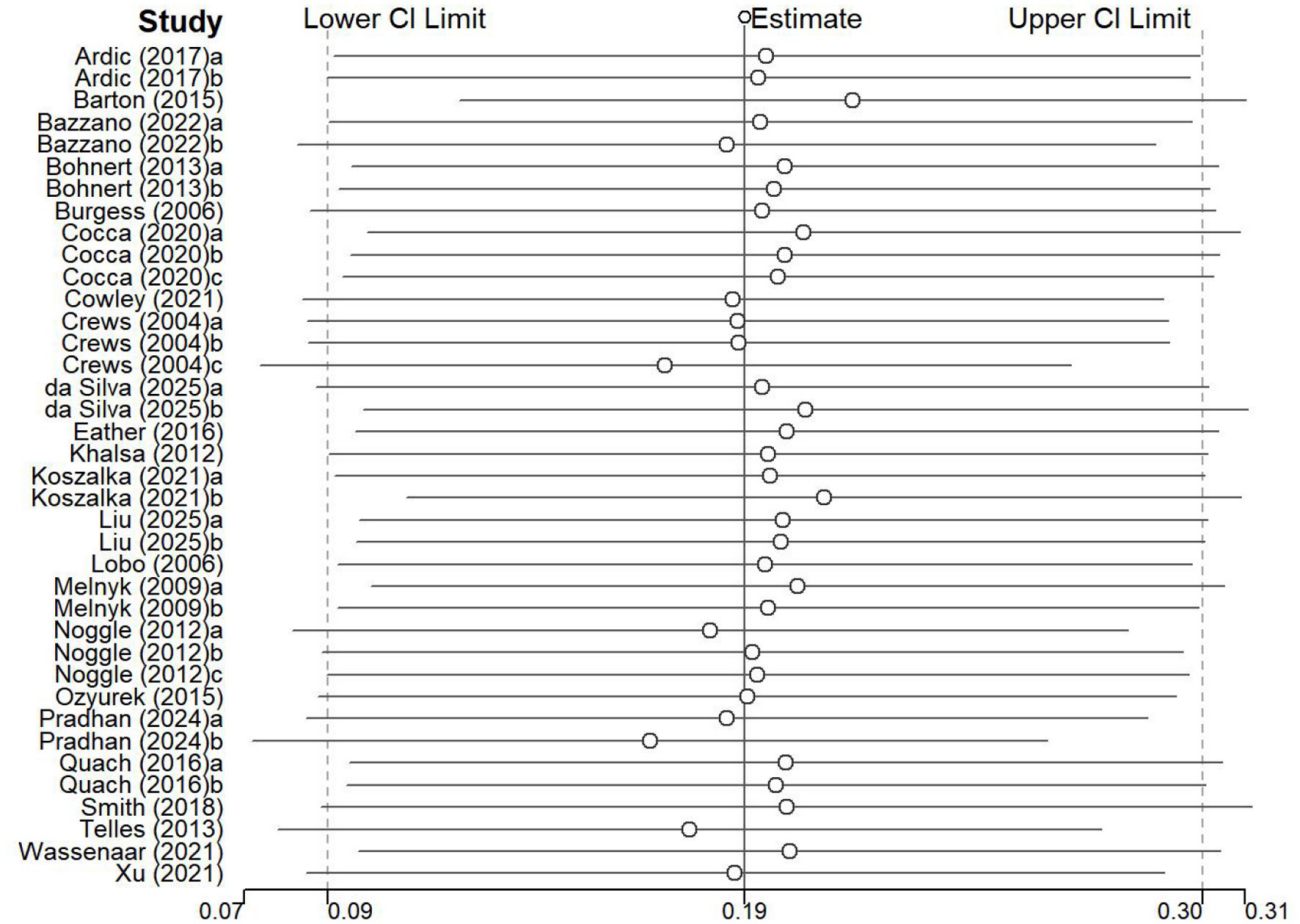
Sensitivity analysis.

To evaluate whether any single study unduly influenced the pooled estimate, we performed a leave-one-out analysis, recalculating the pooled effect after removing one study at a time. The pooled SMD ranged from 0.17 to 0.21 (Figure 4). Across iterations, 95% CIs excluded 0 and overlapped substantially with the primary estimate (SMD = 0.19, 95% CI: 0.09–0.30). For example, excluding a study with a large positive effect (Telles et al., 2013) yielded a pooled SMD of 0.18 (95% CI: 0.08–0.28), whereas excluding a study with an effect in the negative direction (Barton et al., 2015) yielded a pooled SMD of 0.21 (95% CI: 0.11–0.31). These results indicate that the overall conclusion was robust and not driven by any single study.

#### 3.6.2 Comparison of results using different effect models

Given the statistically significant heterogeneity observed in the overall analysis (*I*^2^ = 48%; *p* = 0.0006), the random-effects model was used as the primary model. As a sensitivity analysis, we also calculated the pooled effect using a fixed-effect model. The fixed-effect model yielded a slightly smaller but still statistically significant effect (SMD = 0.15, 95% CI: 0.11–0.19; *p* < 0.00001), which was consistent in direction and significance with the random-effects estimate. These findings support the robustness of the primary result under different model assumptions.

### 3.7 Publication bias assessment

#### 3.7.1 Visual inspection of the funnel plot

**Figure 5:**
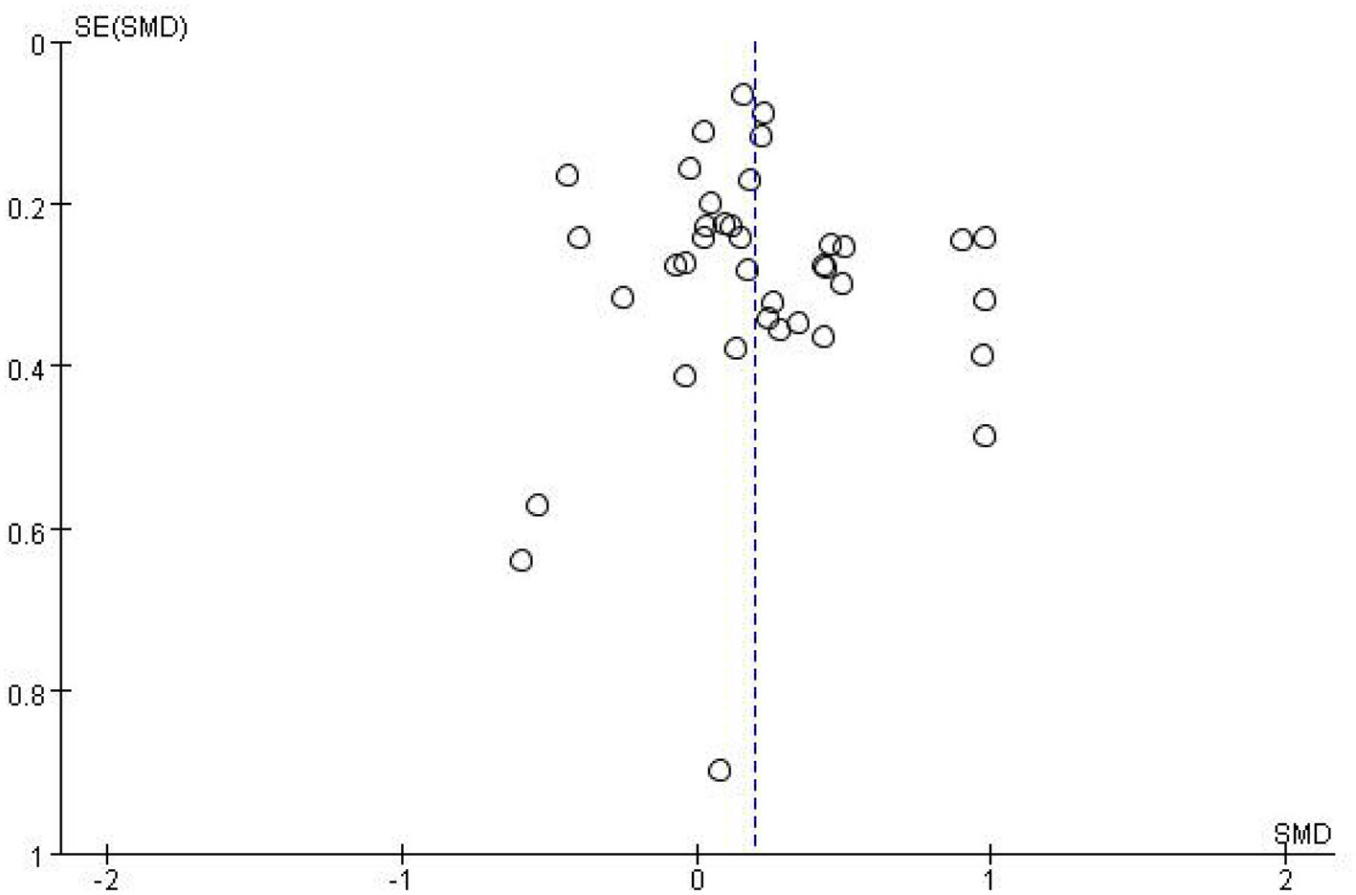
Funnel plot.

To visually assess potential publication bias, we generated a funnel plot (Figure 5) with the standardized mean difference (SMD) on the x-axis and its standard error (SE) on the y-axis. Under the assumption of no substantial publication bias, study effects would be expected to distribute approximately symmetrically around the pooled estimate. Visual inspection suggested that the distribution of study points was generally acceptable but not perfectly symmetrical. Larger, more precise studies clustered relatively close to the pooled estimate (SMD = 0.19), whereas smaller, less precise studies were more dispersed. Overall, the funnel plot suggested minor asymmetry but did not provide clear evidence of substantial publication bias.

#### 3.7.2 Egger's test

To quantify funnel-plot asymmetry, we performed Egger's regression test, in which a statistically significant intercept indicates potential small-study effects. Egger's test yielded an intercept of 0.96 (95% CI: −0.51 to 2.43; *p* = 0.197). Because the intercept was not statistically significant, Egger's test did not provide statistical evidence of funnel-plot asymmetry or substantial publication bias.

#### 3.7.3 Trim-and-fill analysis

Given the slight asymmetry suggested by visual inspection, we applied the trim-and-fill method under a random-effects model to evaluate the potential influence of missing studies. The trim-and-fill analysis did not impute any missing studies (*L* = 0). Accordingly, the adjusted pooled effect remained unchanged (SMD = 0.19, 95% CI: 0.09–0.30), consistent with the primary estimate.

## 4 Discussion

### 4.1 Summary of main findings

This meta-analysis synthesizes the latest available evidence and systematically evaluates the effects of physical activity interventions on mental health in children and adolescents, as well as potential moderators. The quantitative synthesis indicated a statistically significant overall benefit of physical activity for mental health, although the magnitude was small. These findings support physical activity as a universal public health prevention strategy, while also suggesting that its effects when used alone as a clinical intervention may be limited and may need to be complemented by other psychosocial supports in practice.

A key clinically relevant finding is that intervention effects appear to vary substantially by activity type and theoretical orientation. Subgroup analyses suggested that mind–body interventions showed a relatively larger pooled estimate, followed by multicomponent/behavioral interventions, whereas pooled effects for sports/games interventions and aerobic/fitness training were not statistically significant. Collectively, these findings are consistent with a mind–body integration hypothesis, suggesting that interventions explicitly combining physical activity with cognitive and emotional regulation components may provide additional psychological benefits, compared with programs focused primarily on physiological conditioning, skill acquisition, or recreation.

Intervention effects varied across individuals, and baseline psychological risk status emerged as a key moderator: benefits were larger in adolescents with psychosocial risk factors or mild symptoms than in the general population. This pattern highlights the potential value of targeted prevention, suggesting that physical activity interventions may yield greater benefits among those most in need and informing more efficient public health resource allocation. Although the pooled effect estimate was numerically larger in adolescent samples, the test for subgroup differences by age was not statistically significant, indicating that further research is needed. With respect to measurement, pooled estimates varied across measurement types, suggesting that outcome construct may partly contribute to differences in observed effects. Sensitivity analyses supported the robustness of these conclusions, with findings not materially changed by removing individual studies or by alternative model specifications.

### 4.2 Comparison with previous studies and interpretation of innovation points

Our findings not only update the evidence base but also extend prior reviews conceptually and methodologically. This review moves beyond the traditional approach that treats physical activity as a homogeneous intervention. Many earlier reviews pooled diverse activities into a single overall estimate or used coarse classifications based on surface features (Hou et al., 2025). Such approaches can obscure meaningful differences in underlying mechanisms and active components across activity types (Higgins et al., 2019; Nakagawa et al., 2023). Accordingly, we applied a prespecified four-category framework based on theoretical underpinnings and active components: mind–body, multicomponent/behavioral, sports/games, and aerobic/fitness training interventions. Results indicated statistically significant differences across these intervention categories. This framework may help explain inconsistencies in prior findings and shifts the focus from whether physical activity is beneficial to which intervention types are most effective, thereby increasing practical relevance for policy and implementation.

We further identified baseline psychological risk status as a key moderator, extending the current evidence base. Although individual differences are widely acknowledged, many previous reviews did not formally examine or report moderation by baseline risk status (Peng et al., 2022; Cooke, 2024). Our subgroup analyses showed larger benefits among adolescents with psychosocial risk factors or subclinical psychological distress than among the general population. This finding has important public health implications. Under constrained resources, prioritizing structured programs, particularly mind–body interventions, for high-risk groups such as students in low-income school settings or those identified through screening may improve the efficiency of mental health prevention efforts. This offers an empirical rationale for more targeted and equitable delivery of preventive mental health services.

This review also expands the timeliness and breadth of the evidence base. It incorporates recently published randomized controlled trials, including trials targeting neurodevelopmental or psychological risk groups such as attention-deficit/hyperactivity disorder (ADHD). By integrating newer trials, our findings address evidence gaps in earlier reviews and provide an updated basis for current and future practice and policy decisions.

### 4.3 Potential mechanisms and interpretation

The heterogeneous pattern of effects observed in this study may be interpreted at multiple levels, including intervention mechanisms, differences in population responses, and implementation context. The comparatively stronger effects of mind–body interventions may reflect their more direct training of stress regulation and emotion regulation systems. These interventions typically integrate body awareness, breath regulation, and meditation based attentional training (Bianjiang et al., 2025; Yook et al., 2017). From a neurophysiological perspective, breathing practices and meditation have been shown to enhance parasympathetic activity, modulate hypothalamic–pituitary–adrenal (HPA) axis function, and reduce physiological stress responses (Pascoe et al., 2021; Little, 2025). From a cognitive neuroscience perspective, these practices may strengthen regulatory processes involving the prefrontal and limbic systems and improve the efficiency of emotion regulation by repeatedly reorienting attention to present moment experience (Doll et al., 2016; Lutz et al., 2014). Psychologically, they may cultivate nonjudgmental metacognitive skills that support acceptance of current experience, which is relevant to reducing repetitive negative thinking in anxiety and depression (Jackson and Jones, 2025; Wei et al., 2025). Accordingly, mind–body interventions may provide both the general physiological benefits of physical activity and more explicit psychological skill training for managing distress.

The finding that physical activity showed larger effects in risk or trait populations may align with an intervention response potential hypothesis (Li et al., 2025). In the general population, baseline emotional distress may be relatively low, which can limit observable improvement. In contrast, high risk or mildly symptomatic groups may have higher baseline distress and therefore greater room for improvement (Sims et al., 2025; Schmit et al., 2024). They may also have fewer effective emotion regulation strategies at baseline (Sims et al., 2025; Schmit et al., 2024). In this context, physical activity interventions may produce greater benefits when they provide structured routines, opportunities for mastery, social support, and repeated practice of self-regulation. These mechanisms may be particularly relevant for youth with psychosocial risk factors or early psychological symptoms, because the intervention components may directly target difficulties that are already present or emerging in these groups. Accordingly, when interventions, particularly mind–body or multicomponent/behavioral programs, provide these skills, benefits may be more pronounced. This suggests that mental health benefits may not simply increase with greater intervention dose; instead, there may be an individualized window of responsiveness, particularly among adolescents showing early signs of distress. The age subgroup findings should be interpreted cautiously, because the test for subgroup differences was not statistically significant.

The pooled effects of sports/games interventions and aerobic/fitness training were not statistically significant. This finding suggests that simply increasing activity may not be sufficient to improve mental health. In school or community settings, these activities may improve physical fitness, enjoyment, and social interaction, but their psychological effects may be more indirect than those of interventions that explicitly train emotion regulation. For some participants, especially adolescents with lower skill levels, reduced self-confidence, or elevated psychological distress, competitive or performance focused activities may inadvertently introduce performance pressure, social comparison, or competitive anxiety. Such negative experiences may offset potential benefits, such as feelings of achievement or social enjoyment (Ruiz-Ranz and Asín-Izquierdo, 2024; Zhang et al., 2024). These activities may also lack explicit components targeting emotion regulation or cognitive restructuring, and any benefits may be indirect and insufficient to produce durable changes in trait anxiety or internalizing problems detectable with standardized measures (White et al., 2024). This underscores the importance of matching intervention components to participants' psychological needs, rather than focusing solely on increasing duration or intensity.

### 4.4 Research advantages and limitations

This review has several methodological and analytical strengths. The protocol was prospectively registered in PROSPERO, and reporting followed the PRISMA 2020 statement. Study selection, data extraction, and risk of bias assessment were performed independently by two reviewers using design appropriate tools. The inclusion of quasi-experimental studies also broadened the ecological relevance of the evidence by capturing interventions implemented in real-world school and community settings, while the ROBINS-I findings guided a cautious interpretation of their causal contribution. In addition, the prespecified subgroup analyses and theory-based intervention classification framework improved interpretability and provided actionable evidence for intervention design and policy.

Several limitations should be considered. Residual heterogeneity suggests that unmeasured factors, such as intervention dose, implementation fidelity, instructor qualifications, and sociocultural context, may have influenced the results. Measurement differences and limited evidence in some subgroups may have reduced the power to detect moderation. Finally, the lack of longer follow-up in most studies limits conclusions about the durability of mental health benefits.

### 4.5 Implications for practice and policy

Our findings provide an empirical basis for informing the design and implementation of physical activity programs that target mental health in practical settings. At the level of program design, the relatively larger pooled estimates observed for mind–body interventions suggest that modules integrating physical activity with training in emotion regulation skills could be considered within school curricula, after school programs, and community health initiatives (Phan et al., 2022; McCurdy et al., 2024). In contrast, simply expanding traditional physical education or emphasizing competitive or fitness focused training may be less likely to yield meaningful mental health benefits unless explicit psychological skill development components are included (Piñeiro-Cossio et al., 2021; Wang et al., 2025). At the level of policy and resource allocation, our results support targeted prevention strategies to improve mental health promotion and early prevention. Benefits appeared larger among adolescents with elevated psychological risk. Accordingly, resources and services could be prioritized for schools and communities with concentrated risk, including those in areas with low socioeconomic status or limited access to mental health services. In such settings, implementing structured mind–body programs supported by evidence may serve as a feasible approach for mental health promotion and early prevention (Le et al., 2021; Kronenberg et al., 2025). Achieving targeted implementation of high quality will likely require sustained collaboration across sectors and disciplines. Such collaboration can ensure that programs are grounded in sport science while embedding psychological components supported by evidence, and can be iteratively refined through rigorous evaluation to bridge the gap between research and practice.

### 4.6 Suggestions for future research

Building on the findings and limitations of this review, future research could advance understanding and strengthen evidence-based practice in several areas. First, randomized trials focused on mechanisms should move beyond evaluations that treat interventions as a black box by prespecifying and measuring key mediators that explain how physical activity interventions influence mental health. Second, comparative effectiveness studies are needed to directly compare different intervention types within the same population and setting to estimate relative benefits. Third, follow-up assessments over longer periods and at multiple time points would clarify the durability of mental health effects beyond outcomes measured immediately after intervention. Fourth, research should examine acceptability, feasibility, and effectiveness across diverse sociocultural contexts, including appropriate cultural adaptations to intervention content and delivery, particularly in settings outside Western contexts or settings with limited resources. Finally, improving reporting quality and transparency in primary studies, particularly by providing detailed descriptions of intervention components, theoretical rationale, implementation procedures, and participant characteristics, would facilitate more informative evidence synthesis and help explain heterogeneity.

## 5 Conclusion

Our findings indicate that physical activity interventions were associated with small but statistically significant improvements in mental health among children and adolescents. Benefits varied by intervention type and baseline psychological risk status, with relatively larger pooled estimates observed for mind–body interventions and for youth with elevated psychological risk; however, these subgroup findings should be interpreted cautiously.

From a practical and clinical perspective, structured physical activity programs may serve as accessible and low burden strategies for mental health promotion and early prevention, particularly in schools, communities, and youth health services. Programs that integrate physical activity with psychological regulation components may be especially useful for children and adolescents with psychosocial risk factors or mild psychological symptoms. These findings support the targeted implementation of structured physical activity interventions as a complementary approach to existing psychosocial and mental health support.

## Data Availability

The original contributions presented in the study are included in the article/Supplementary material, further inquiries can be directed to the corresponding author.
