# Supplementary material for: Effects of physical activity interventions on mental health in children and adolescents: a systematic review and meta-analysis of intervention type and baseline risk as moderators
Source: Front Psychol. 2026 May 29;17:1825603. doi: 10.3389/fpsyg.2026.1825603 (PMC13259663; doi:10.3389/fpsyg.2026.1825603)
Supplement: Supplementary file 1 [file Supplementary_File_1.docx]

Supplementary Table S1. Detailed Search Strategies

| Source : PubMed |
| --- |
| Search strategy : ("Child"[MeSH] OR "Adolescent"[MeSH] OR child*[Title/Abstract] OR adolescen*[Title/Abstract] OR youth[Title/Abstract] OR teen*[Title/Abstract] OR student*[Title/Abstract]) **AND** ("Exercise"[MeSH] OR "Physical Education and Training"[MeSH] OR "Yoga"[MeSH] OR "physical activity"[Title/Abstract] OR "physical exercise"[Title/Abstract] OR exercise[Title/Abstract] OR "exercise training"[Title/Abstract] OR "exercise intervention"[Title/Abstract] OR "physical education"[Title/Abstract] OR sport*[Title/Abstract] OR yoga[Title/Abstract] OR "tai chi"[Title/Abstract] OR qigong[Title/Abstract] OR dance[Title/Abstract] OR aerobic[Title/Abstract] OR fitness[Title/Abstract] OR HIIT[Title/Abstract] OR "high-intensity interval training"[Title/Abstract]) **AND** ("Anxiety"[MeSH] OR "Anxiety Disorders"[MeSH] OR "Depression"[MeSH] OR "Depressive Disorder"[MeSH] OR anxiety[Title/Abstract] OR depression[Title/Abstract] OR depressive[Title/Abstract] OR "mental health"[Title/Abstract] OR internalizing[Title/Abstract] OR internalising[Title/Abstract] OR distress[Title/Abstract] OR "psychological distress"[Title/Abstract]) **AND** (randomized[Title/Abstract] OR randomised[Title/Abstract] OR trial[Title/Abstract] OR intervention[Title/Abstract] OR "controlled trial"[Title/Abstract] OR "quasi-experimental"[Title/Abstract] OR "quasi experimental"[Title/Abstract]) **NOT** (animals[MeSH] NOT humans[MeSH]) |
| Filters / Limits : Search fields: MeSH and title/abstract |
| Records identified : 3,245 |
| Source : Embase |
| Search strategy : ('child'/de OR 'adolescent'/de OR child*:ti,ab OR adolescen*:ti,ab OR youth:ti,ab OR teen*:ti,ab OR pupil*:ti,ab OR schoolchild*:ti,ab OR 'school-aged':ti,ab OR 'school age':ti,ab OR 'young people':ti,ab) **AND** ('physical activity':ti,ab OR 'physical exercise':ti,ab OR exercise:ti,ab OR 'exercise training':ti,ab OR 'exercise intervention':ti,ab OR 'physical education':ti,ab OR yoga:ti,ab OR 'tai chi':ti,ab OR qigong:ti,ab OR dance:ti,ab OR aerobic:ti,ab OR hiit:ti,ab OR 'high intensity interval training':ti,ab OR 'high-intensity interval training':ti,ab) **AND** (anxiety:ti,ab OR depression:ti,ab OR depressive:ti,ab OR 'mental health':ti,ab OR internalizing:ti,ab OR internalising:ti,ab OR distress:ti,ab OR 'psychological distress':ti,ab) **AND** (randomized:ti,ab OR randomised:ti,ab OR trial:ti,ab OR intervention:ti,ab OR 'controlled trial':ti,ab OR 'quasi experimental':ti,ab OR 'quasi-experimental':ti,ab) **AND** [humans]/lim |
| Filters / Limits : Search fields: Emtree terms and title/abstract; limited to humans |
| Records identified : 3,812 |
| Source : PsycINFO |
| Search strategy : (child* OR adolescen* OR youth OR teen* OR pupil* OR schoolchild* OR "school-aged" OR "school age" OR "young people" OR school*).ti,ab. **AND** ("physical activity" OR "physical exercise" OR exercise OR "exercise training" OR "exercise intervention" OR "physical education" OR sport* OR yoga OR "tai chi" OR qigong OR dance OR aerobic OR fitness OR HIIT OR "high intensity interval training" OR "high-intensity interval training").ti,ab. **AND** (anxiety OR depression OR depressive OR "mental health" OR internalizing OR internalising OR distress OR "psychological distress").ti,ab. **AND** (random* OR randomized OR randomised OR trial* OR intervention* OR "controlled trial" OR "quasi experimental" OR "quasi-experimental").ti,ab. |
| Filters / Limits : Search fields: title and abstract; via Ovid. |
| Records identified : 1,905 |
| Source : Cochrane CENTRAL |
| Search strategy : (child* OR adolescen* OR youth OR teen* OR pupil* OR schoolchild* OR "school-aged" OR "school age" OR "young people"):ti,ab,kw AND ("physical activity" OR "physical exercise" OR "exercise training" OR "exercise intervention" OR "physical education" OR yoga OR "tai chi" OR qigong OR dance OR aerobic OR HIIT OR "high intensity interval training" OR "high-intensity interval training"):ti,ab,kw AND (anxiety OR "anxiety disorder" OR depression OR internalizing OR internalising OR "psychological distress"):ti,ab,kw AND (random* OR trial* OR "controlled trial" OR "quasi experimental" OR "quasi-experimental"):ti,ab,kw |
| Filters / Limits : Search fields: title, abstract, and keywords; trials/CENTRAL records only |
| Records identified : 1,021 |
| Source : Web of Science |
| Search strategy : TS=(child* OR adolescen* OR youth OR teen* OR pupil* OR schoolchild* OR "school-aged" OR "school age" OR "young people") **AND** TS=("physical activity" OR "physical exercise" OR "exercise training" OR "exercise intervention" OR "physical education" OR sport* OR yoga OR "tai chi" OR qigong OR dance OR aerobic OR HIIT OR "high intensity interval training" OR "high-intensity interval training") **AND** TS=(anxiety OR depression OR depressive OR "mental health" OR internalizing OR internalising OR distress OR "psychological distress") **AND** TS=(random* OR trial* OR "controlled trial" OR "quasi experimental" OR "quasi-experimental") |
| Filters / Limits : Search field: Topic |
| Records identified : 1,987 |
| Source : SPORTDiscus |
| Search strategy : (TI (child* OR adolescen* OR youth OR teen* OR pupil* OR schoolchild* OR "school-aged" OR "school age" OR "young people") OR AB (child* OR adolescen* OR youth OR teen* OR pupil* OR schoolchild* OR "school-aged" OR "school age" OR "young people")) **AND** (TI ("physical activity" OR "physical exercise" OR "exercise training" OR "exercise intervention" OR "physical education" OR sport* OR yoga OR "tai chi" OR qigong OR dance OR aerobic OR HIIT OR "high intensity interval training" OR "high-intensity interval training") OR AB ("physical activity" OR "physical exercise" OR "exercise training" OR "exercise intervention" OR "physical education" OR sport* OR yoga OR "tai chi" OR qigong OR dance OR aerobic OR HIIT OR "high intensity interval training" OR "high-intensity interval training")) **AND** (TI (anxiety OR depression OR depressive OR "mental health" OR internalizing OR internalising OR distress OR "psychological distress") OR AB (anxiety OR depression OR depressive OR "mental health" OR internalizing OR internalising OR distress OR "psychological distress")) **AND** (TI (random* OR trial* OR intervention* OR "controlled trial" OR "quasi experimental" OR "quasi-experimental") OR AB (random* OR trial* OR intervention* OR "controlled trial" OR "quasi experimental" OR "quasi-experimental")) |
| Filters / Limits : Search fields: title and abstract; via EBSCOhost |
| Records identified : 488 |
| Source : ClinicalTrials.gov |
| Search strategy : Condition/disease: anxiety OR depression OR "mental health" OR internalizing OR distress OR "psychological distress"; Other terms: child OR children OR adolescent OR adolescents OR youth OR teen OR teenager OR teenagers; Intervention/treatment: "physical activity" OR exercise OR "exercise therapy" OR sport OR sports OR yoga OR "tai chi" OR qigong OR dance OR aerobic OR fitness OR HIIT OR "high-intensity interval training" OR "physical education" |
| Filters / Limits : Study type: Interventional studies; Study status: All studies; Age: 3–19 years |
| Records identified : 753 |
| Source : WHO ICTRP |
| Search strategy : Condition: anxiety OR depression OR "mental health" OR internalizing OR distress OR "psychological distress"; Intervention: "physical activity" OR exercise OR "exercise therapy" OR sport OR sports OR yoga OR "tai chi" OR qigong OR dance OR aerobic OR fitness OR HIIT OR "high-intensity interval training" OR "physical education" |
| Filters / Limits : Clinical trials in children; recruitment status: all; phases: all |
| Records identified : 274 |
| Source : ProQuest Dissertations & Theses Global |
| Search strategy : Abstract: ("physical activity" OR exercise OR "exercise training" OR "exercise intervention" OR "physical education" OR yoga OR "tai chi" OR qigong OR dance OR aerobic OR HIIT) **AND** Abstract: (child OR children OR adolescent OR adolescents OR youth OR teen OR teenagers) **AND** Abstract: (anxiety OR depression OR "mental health" OR internalizing OR distress OR "psychological distress") **AND** Abstract: (intervention OR trial OR randomized OR randomised OR program OR programme OR training) |
| Filters / Limits : Search field: Abstract |
| Records identified : 98 |
| Source : Citation searching |
| Search strategy : Manual citation searching of the reference lists of the 24 included studies and relevant systematic reviews/meta-analyses published within the past five years. |
| Filters / Limits : Potentially relevant original intervention studies were identified according to the review eligibility criteria. |
| Records identified : 62 (38 from included studies; 24 from relevant reviews/meta-analyses) |


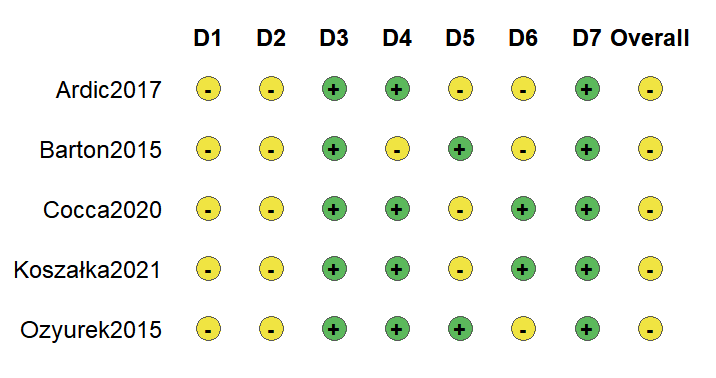


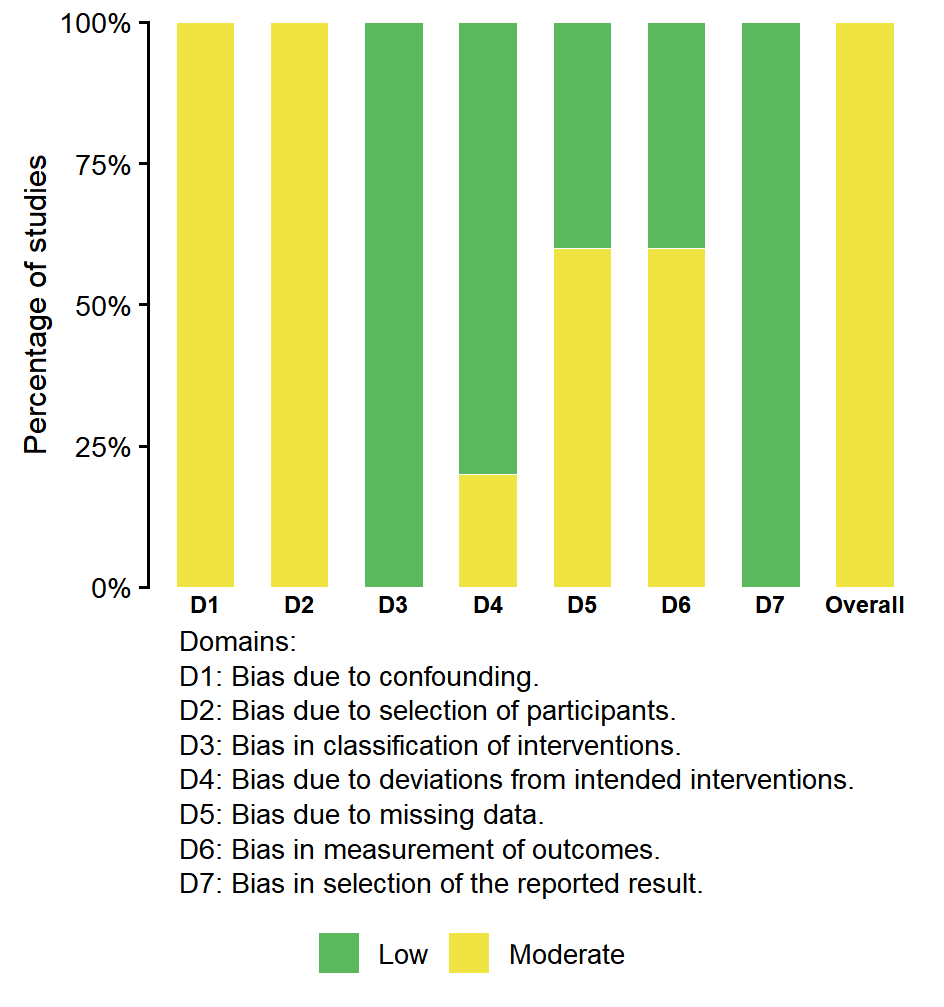


Supplementary Figure 1

ROBINS-I.


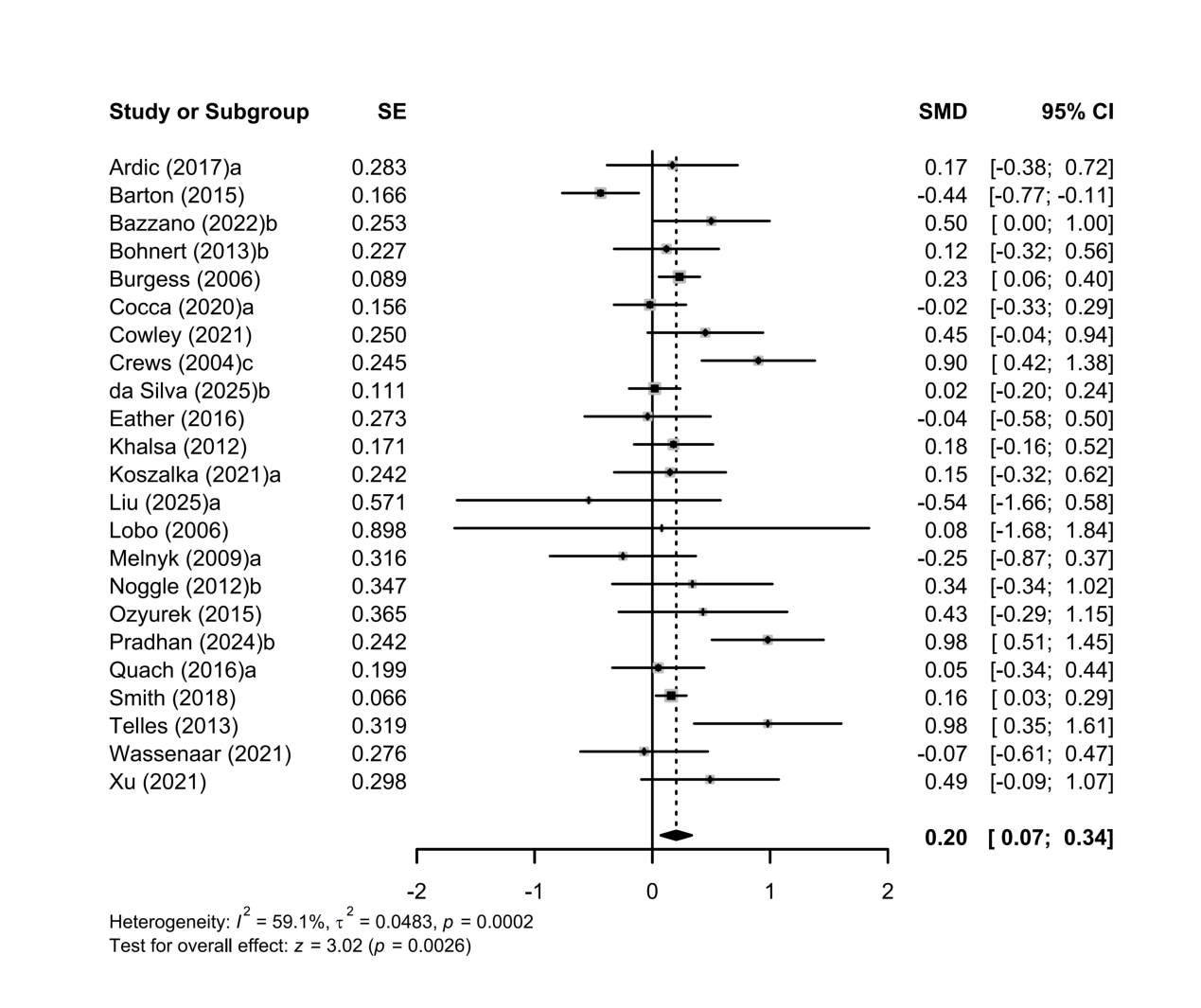


Supplementary Figure 2

Forest plot of the one-estimate-per-study sensitivity analysis.


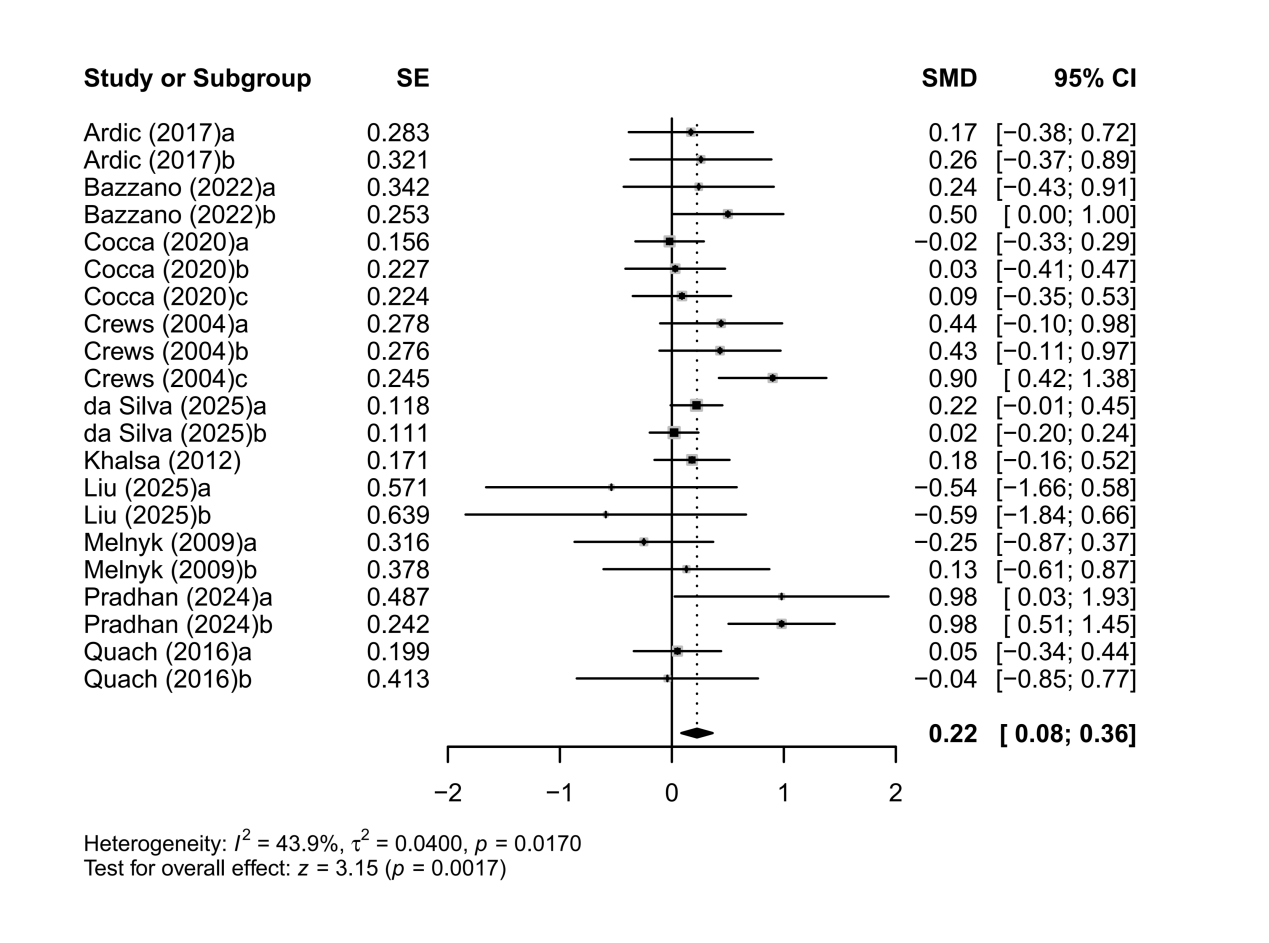


Supplementary Figure 3

Forest plot of the sensitivity analysis restricted to anxiety-specific scales.
